# The 13 item Family Support Scale: Reliability and validity of the Greek translation in a sample of Greek health care professionals

**DOI:** 10.1186/1447-056X-10-3

**Published:** 2011-04-13

**Authors:** Athanasios Tselebis, Tania Anagnostopoulou, Dionisios Bratis, Aikaterini Moulou, Alexia Maria, Christos Sikaras, Ioannis Ilias, Athanasios Karkanias, Georgios Moussas, Nikolaos Tzanakis

**Affiliations:** 1 Sotiria General Hospital, Athens, Greece; 2 Hellenic Institute of Psychology & Health, Thessaloniki, Greece; 3 Department of Endocrinology, "Elena Venizelou" Hospital, Athens, Greece; 4 Dept of Social Medicine, Medical School, University of Crete, Heraklion, Greece

## Abstract

**Background:**

The Julkunen Family Support Scale aims to record the sense of support that a subject receives from the members of his family. The object of the present study was to investigate the reliability and to assess the validity of the Greek translation of the Julkunen Family Support Scale in Greek health care professionals in a public general hospital.

**Methods:**

In order to determine the indicator of validity of content we addressed nine expert professionals and one sociologist, asking them to evaluate how much relevant to the sense of familial support are the items of the questionnaire. Additionally, to assess reliability we used a sample of health care professionals.

**Results:**

There was agreement among experts for the validity of content. Cronbach's alpha for the total items was 0.820, pointing to high validity. Only replacing item four could increase the scale's validity, but without significant differences.

**Conclusions:**

The scale, in its Greek version, appears to be a brief and reliable tool that can be used for inpatients, in clinics as well as in epidemiologic studies of received family support.

## Background

In the international bibliography of health psychology, many studies have attempted to delineate the relationship between the input in family support of patients with acute or chronic disease or their level of anxiety or depression [1], as well as the effect of this support in the confrontation of illness [2,3]. Simultaneously, other research studies have focused in the cross-correlation of degrees of familial support with the presence of burnout in health professionals and, particularly, in nurses [4]. The Julkunen Family Support Scale (Julkunen J & Greenglass ER: The family support scale, submitted) has been conceived in Finland and aims to record the sense of support that a subject receives from the members of his family (with whom he/she lives). The scale is constituted by 13 items, which are answered on a Likert scale, ranging from 1 ("I disagree a lot") to 5 ("I agree a lot"). Examples of subjects of particular items are: "My family supports me in each my effort" (item 1) and "there is no benefit in speaking about your daily difficulties at home" (item 4). At the same time, the examinee is asked to record the number of members of his family that live together. Seven of thirteen items (items 4, 5, 6, 8, 9, 10 and 11) are graded inversely. The scale is self-answered and it is not recommended to be given to individuals that live alone, since all of the items focus on the interrelations of individuals that live together. High scores correspond to an increased sense of family support. For every subject the scale does not aim to examine objectively the family support that the individual receives, but rather the sense that he/she has of how much he/she is supported by the persons with whom he/she lives together. The particular scale has been translated in Greek (T. Anagnostopoulou, Hellenic Institute of Psychology & Health, Thessaloniki) and has been used in a number of research works in Greece [5-11]. In these studies the measurement of perceived family support focused on the particularities that characterize interpersonal relations in Greek society and, more specifically, in the Greek family. The Greek family is characterized by very close-knit relationships; it constitutes often the basic source for receiving support and feedback, while also appearing to function protectively against stressful events. The lack of a family network or the existence of disturbed relations inside the narrow family environment appears to relate with higher stress levels [5-7] and depression [10]. Furthermore, a high degree of sense of received family support appears to be linked with better adaptation and confrontation in the case of patients with chronic diseases [8,9]. It is remarkable that in relevant studies the Family Support Scale presents systematically negative cross-correlations with Beck's depression scale (Beck Depression Inventory, BDI), as well as with Spielberger's anxiety scale (State Trait Anxiety Inventory, STAI). The aim of the present study was to investigate the reliability and assess the validity of the Greek translation of the Family Support Scale in Greek health care professionals in a public general hospital.

## Methods

The scale was translated and back translated in Greek by two English speaking psychologists.

To determine the indicator of validity of content we addressed nine expert health professionals (3 psychiatrists, 3 psychologists and 3 nurses) and a sociologist, asking them to evaluate how much relevant to the sense of familial support that somebody feels is the total of the items in the questionnaire. The scale of marking was from 1 "not relevant" to 4 "absolutely relevant"

To asses reliability we used a sample of health care professionals, chosen randomly from workers in one of the bigger general hospitals in Athens, Greece. Overall, 165 workers were asked to participate in the study, 35 of whom were excluded either after declaring that they live alone them or after denying to participate in the study. Of the 130 final participating workers (40 men and 90 women) age and level of education, were recorded, followed by filling in the Family Support Scale. The directions that were given to all participants were as follows: "The questions that follow refer to your family. We ask that you answer each question putting in a circle the number that describes better your family (the individuals with whom you live together)". The time for answering the questionnaire did not exceed 4 minutes.

From the whole sample 28 subjects (7 men and 21 women) were randomly selected to participate in a 30-minute-long personal interview with a psychologist; the latter noted on a scale from 1 (lowest) to 5 (highest) each examinee's perception of family support. The factors that were evaluated in the interview were related to the feelings of tension or any intense arguments that each examinee experienced within his/her home, along with the support that he/she considered to have received in making personal or professional plans as well as whether his/her return home was accompanied by a sense of comfort and rest.

Regarding the scale's reliability, this was assessed with Cronbach's alpha (i.e. a measure of internal consistency). The total alpha coefficient if a given item is deleted was also assessed. Further reliability testing was also implemented with split-half reliability measures (also referred to as internal consistency reliability). These are measures of estimation based on the correlation of two equivalent forms of the scale. More in detail, the Spearman-Brown coefficient was calculated (using samples of unequal length that give the reliability estimate assuming unequal numbers of items in each set; this is necessary in a battery with a total of 13 items). The Guttman split-half reliability coefficient was also calculated (this an adaptation of the Spearman-Brown coefficient that does not require equal variances between the two split forms).

Regarding the interview-based validity leg of the study, comparisons of the subjects' gender (with the Chi-square test) and of age, years of education and Family Support scale (with Student's t-test) were done. Pearson's correlation of the interview-based family support scores and Family Support Scale scores of the administered test was also calculated.

## Results

### Content validity

There was agreement among experts for the validity of content of the questionnaire in its entirety. Three experts considered it "very relevant" and 7 "absolutely relevant".

### Reliability

The age of the study subjects (mean ± SD) 39.23 ± 9.48 and years of education (mean ± SD) 13.35 ± 2.06 did not differ statistically significant between male and female (student t-test p > 0.05). Family support was higher in men (mean ± SD) 50.8 ± 8.6 compared to women (mean ± SD) 47 ± 9.8, (student t-test p < 0.05). Family Support Scale score was not correlated with years of education or age for the whole sample or for each gender examined separately (Person's correlation, p > 0.05, r: 0.010).

The reliability of the questionnaire's 13 items was assessed with Cronbach's alpha and was verified after splitting the sample (Guttmann's "split-half"). More in detail, Cronbach's alpha for the 13 items was 0.820 (table 1), pointing to high validity [12]. Each item's correlation with the entire test was not high (but only items 3,4,8 and 12 were lower than 0.40) nevertheless isolated deletion of each item provided alpha coefficients that fell within a narrow - and acceptable - range from 0.798 to 0.818 [13]. Only replacing item 4 could increase the scale's validity, without, however, important differences. Thus it was chosen that it should not be modified (table 1). Examining the reliability of the scale vis-à-vis gender, alpha was 0.821 for men and 0.821 for women.

**Table 1:** Cronbach's alpha reliability analysis statistics

Family Support Item No	Item's correlation with the entire set	Alpha if item is deleted
1	0.541	0.803
2	0.498	0.807
3	0.334	0.817
4	0.250	0.825
5	0.494	0.805
6	0.571	0.798
7	0.497	0.806
8	0.362	0.818
9	0.539	0.801
10	0.463	0.808
11	0.566	0.799
12	0.322	0.817
13	0.632	0.798

The unequal length Spearman-Brown coefficient (with 6 items in one group and 7 items in the other group) was 0.819. Guttman's "split-half" coefficient was 0.818, confirming the good reliability of the scale.

### Interview-based validity

The subjects that were interviewed did not differ in age (mean ± SD) 39 ± 9.8 vs 39 ± 9.5 years; Student t-test p > 0.05), education level (yrs) (mean ± SD) 13 ± 2 vs 13.4 ± 2.06; Student t-test p > 0.05), Family Support Scale score (mean ± SD): 48.7 ± 9.8 vs 48.3 ± 9.9; Student t-test p > 0.05) or gender (Chi-square p > 0.05) from the rest of the sample.

This group had a mean ( ± SD) interview-based family support score of 3.4 ± 1.3. Men had higher scores compared to women (4.1 ± 0.70 vs 3.09 ± 1.1; Student t-test p < 0.05). A strong positive correlation was noted between interview-based family support scores and Family Support Scale scores (Pearson Correlation, r = 0.750, p < 0.001).

## Discussion

In the present work the Greek version of the Family Support Scale shows important reliability and validity characteristics. The degree of sense of familial support does not appear to be influenced from factors such as age or years of education.

The men studied appear to enjoy a higher sense of family support. Regarding gender, however, more extensive investigation is required, since this finding likely reflects formal characteristic traits of Greek society vis-à-vis formal social roles that the two sexes are called to undertake.

Conceptual validity of the construct is confirmed by studies in different populations [5,7,8]: they show a negative cross-correlation between the Family Support Scale and the BDI depression scale [14,15] as well as the STAI [16,17]. Results of these studies agree with other research works [18-20], according to which, the lack of feeling of familial support constitutes an anxiety- and depression-generating factor, while the presence of feeling of family support can improve the corresponding symptomatology.

In patients with chronic somatic disease, as diabetes mellitus is, the role of family support has been studied extensively [8,9]: an important correlation has been noted with the course of disease, a fact that it is likely to imply better conformity with medical advice and therapy.

## Conclusions

The Family Support Scale, in its Greek version, appears to be a brief and reliable tool that can be used for inpatients, in clinics as well as in epidemiologic studies of received family support.

## Competing interests

The authors declare that they have no competing interests.

## Authors' contributions

**AT **and **TA **conceived the paper; **AT**, **DB **and **II **carried out the statistical analysis and drafted the paper; **AikM**, **AM **and **CS **performed the scale, collected data and helped drafted the paper; **AK**, **GM **and **NT **supervised the study.

All authors read and approved the final manuscript.
